# Intra-platform comparison of 25-mer and 60-mer oligonucleotide Nimblegen DNA microarrays

**DOI:** 10.1186/1756-0500-6-43

**Published:** 2013-02-04

**Authors:** Stephane Fenart, Malika Chabi, Sophie Gallina, Rudy Huis, Godfrey Neutelings, Nathalie Riviere, Brigitte Thomasset, Simon Hawkins, Anca Lucau-Danila

**Affiliations:** 1 Université Lille Nord de France, Lille 1, UMR INRA 1281, SADV, Villeneuve d’Ascq cedex, F- 59650, France; 2 Roche Diagnostics France, 2, Avenue du Vercors, Meylan, 38242, France; 3 Université Lille Nord de France, Lille 1, GEPV, Laboratoire de Génétique & Évolution des Populations végétales, CNRS UMR 8198, Villeneuve d’Ascq cedex, F- 59650, France; 4 BIOGEMMA, Z.I, du Brezet, 8 rue des Frères Lumières, Clermont-Ferrand cedex 2, 63028, France; 5 UMR CNRS 6022, GEC, Université de Technologie de Compiègne, BP 20529, Compiègne cedex, 60205, France

**Keywords:** Nimblegen, DNA arrays, Gene expression

## Abstract

**Background:**

We performed a Nimblegen intra-platform microarray comparison by assessing two categories of flax target probes (short 25-mers oligonucleotides and long 60-mers oligonucleotides) in identical conditions of target production, design, labelling, hybridization, image analyses, and data filtering. We compared technical parameters of array hybridizations, precision and accuracy as well as specific gene expression profiles.

**Results:**

Comparison of the hybridization quality, precision and accuracy of expression measurements, as well as an interpretation of differential gene expression in flax tissues were performed. Both array types yielded reproducible, accurate and comparable data that are coherent for expression measurements and identification of differentially expressed genes. 60-mers arrays gave higher hybridization efficiencies and therefore were more sensitive allowing the detection of a higher number of unigenes involved in the same biological process and/or belonging to the same multigene family.

**Conclusion:**

The two flax arrays provide a good resolution of expressed functions; however the 60-mers arrays are more sensitive and provide a more in-depth coverage of candidate genes potentially involved in different biological processes.

## Background

Technologies for performing genome-wide expression analyses have rapidly multiplied in recent years and different cross-platform studies have focused on target type, target production and design, labelling or hybridization protocols [1-4] as well as mathematical approaches [5-8]. Despite the tremendous progress in Next Generation Sequencing (NGS) technology and the increasing use of RNAseq approaches, different microarray platforms continue to generate large amounts of high quality expression data for a wide range of animal and plant species and are extensively applied in medical decision-making research. In general, arrays can contain oligonucleotide probes of 25, 30, 40, 50, 60, 65, 70–80 bases in length [9]. For example, *in situ* synthesized arrays for human, mouse, yeast, rat, Arabidopsis, Drosophila, *C. elegans*, zebrafish and other species, can use 25-mers probes (Affimetrix platform), 50-mers probes (Illumina platform), 60-mers probes (Agilent platform), and 50-75-mers probes (Nimblegen platform) [10].

Since 1999 Roche NimbleGen provides high-density arrays for advanced gene expression analysis, synthesized by digital light processing and rapid, high-yield photochemistry using Maskless Array Synthesis (MAS) technology. These arrays present the advantage of a custom design allowing specification of the regions of interest or the targeted probes for a tailored array solution in any organism (http://www.nimblegen.com). Nimblegen 25-mers arrays were successfully used in gene expression analyses in bacteria [11], yeast [12], and human [13]. Nimblegen 36-mers arrays were used in rice [14], and 50- to 75-mers were also used in bacteria [15,16], zebrafish [17], *Mus musculus*[18], human [19], alga [20], poplar [21], Arabidopsis [22], rice [23] and many other species.

We have recently developed a flax high-density oligo-microarray platform using Nimblegen technology [24]. Flax (*Linum usitatissimum* L.) is one of mankind’s oldest cultivated plants and is grown for both its cellulose-rich fibers and for its seeds rich in alpha linolenic acid (ALA, C18:3) [25]. Our current flax platform is based upon a uniplex 385K system consisting of 8 short (25-mers) oligonucleotides per unigene and a total of 48,021 unigenes per slide. This platform represents the first high-density flax microarray system and is currently providing extremely useful biological information [24,26]. Nevertheless, we wanted to know whether adifferent design based upon long oligonucleotides (60-mers) would improve the performances of our gene expression analyses and consequently increase the yield of meaningful biological information. In order to do this we compared two categories of flax target probes: short (25-mers) oligonucleotides and long (60-mers) oligonucleotides in identical conditions of target production, design, labelling, hybridization, image analyses, and data filtering. This comparison was realized with two different flax samples and each RNA sample was used for the two categories of arrays. Experiments were realized in order to discriminate specific gene expression profiles of two different flax tissues, and results were cross-validated using an independent method (qRT-PCR). In this paper technical parameters of array hybridizations are compared and their relevance for the generation of biologically useful information are discussed.

## Results and discussion

The Nimblegen array system is based upon the hybridization of a single labelled sample (derived from RNA), followed by one-channel detection. The intensity of the hybridization signal is then used to determine target concentration. We used two contrasted samples, one from flax inner stem tissue and the other from the outer stem tissue (Additional file 1). These two tissues are easily separated without cross-contamination as previously demonstrated [27].

Three independent hybridizations were performed for each sample using the two array types (25-mers and 60-mers). After verifying the hybridization quality for all experiments the results obtained using the 2 arrays were compared by evaluating the precision and accuracy of expression measurements; a sub-list of 9 genes was used for the comparison of microarray data and qRT-PCR data.

### Hybridization quality

Our results (GSE37980) showed that all probes present on the two types of array were capable of hybridizing successfully (signal>background). The sensitivity of both array types was demonstrated by the wide signal dynamic range obtained (log2 values of 6 to 15 for 25-mers arrays and 4 to 15 for 60-mers arrays). Hybridization quality was verified using experimental metrics reports (NimbleScan v2.5) as recommended by Roche/Nimblegen. This program generates summary statistics (interquartile density, signal range, uniformity mean, uniformity CV (coefficient of variation), number of empty features on the array, mean empty, the number of random control features present on the array, mean random). All metrics were within the recommended value range indicating that hybridization quality was satisfactory for all experiments and any potential artifacts during hybridization were registered for both array types and for all samples. Raw expression data on all flax hybridization experiments were normalized through RMA (Robust Multi-array Average) algorithms included in the NimbleScan software and 46,589 common targets on both array types were taken into account for further comparisons.

### Comparison of the precision of expression measurements

Precision (also called reproducibility or repeatability) represents the degree to which repeated measurements of the same sample hybridization will show the same or similar results [28]. In order to compare the precision of the measurements derived from each array type we used the following criteria: 1) the distribution of inter-slide variation measures; 2) inter-slide correlation of expression profiles.

**Figure 1:**
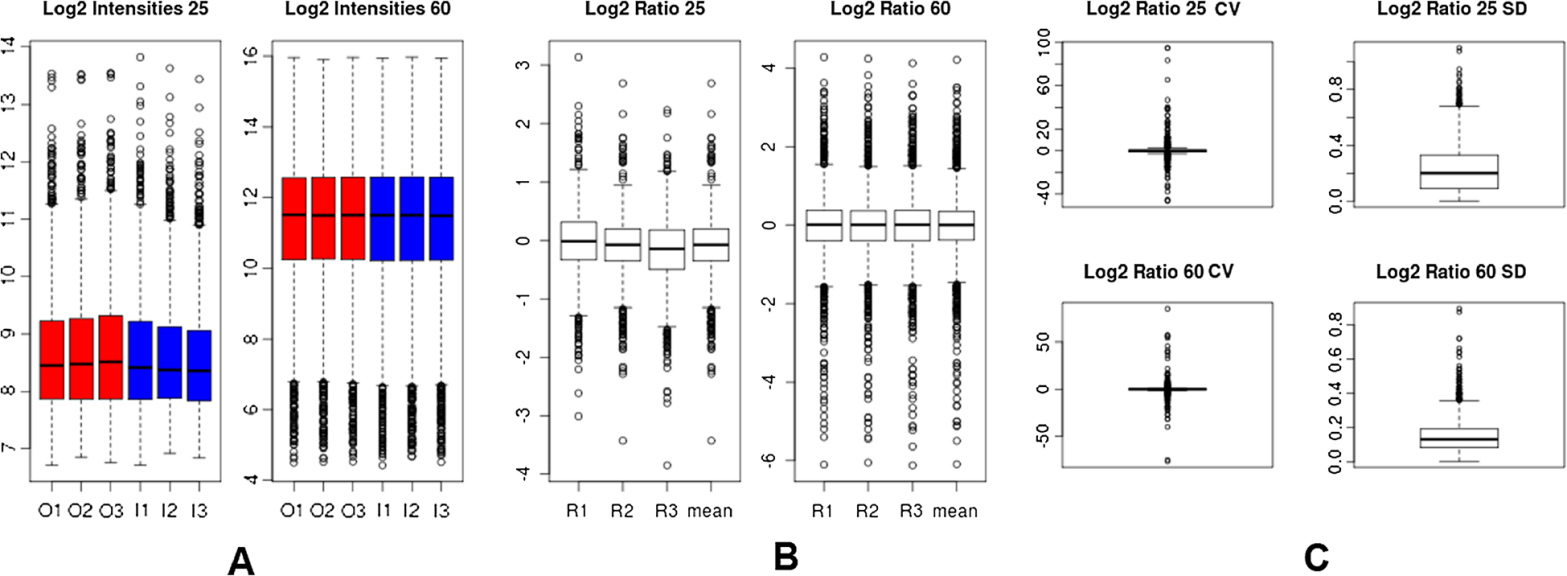
**Boxplots of the distributions of inter-slide hybridization signal intensities and log2 ratios. A**: hybridization signal intensities for all experiments in the two array types (I-inner tissues; O-outer tissues); **B**: expression measurements between each pair-wise combination of inner *vs.* outer tissues (R1, R2, R3 - replicates 1, 2, 3); **C**: distribution of the average inter-slides variations measured as the coefficient of variation (CV) or standard deviation (SD) of log2 ratios for 25-mers (25) and 60-mers flax Nimblegen arrays (60). Data from both array types are highly reproducible, nevertheless, the 60-mers arrays presented globally higher signal intensities and lower variation measures compared to 25-mers array type.

Comparison of the hybridization signal intensities for all experiments in the two array types (Figure 1A), and of expression measurements between each pair-wise combination of inner *vs.* outer tissues (Figures 1B and 1C), show that the data from both array types is highly reproducible. Nevertheless, the 60-mers arrays presented globally higher signal intensities and lower variation measures compared to 25-mers array type as shown by the coefficient of variation (CV) and standard deviation (SD) values for both array types (Figure 1C).

Variations in signal intensities of probes corresponding to different regions of the same mRNA target have previously been observed [29,30], and highly sequence dependent [31-33]. The hybridization efficiency between a probe and its targets is determined by the balance between the binding strength of the probe-target duplex and the formation of probe-probe dimers and secondary structures in either probes or targets [34,35]. The duplex melting temperature is generally considered as one of the most popular measures in the evaluation of microarray probes. It gives the temperature at which half of all probes form a duplex with their target while the other half are unbound, assuming a simple two state transition [34]. General thermodynamic models of probe-target hybridization have also recently been used to compare 25- and 45 to 75-mers tiling Nimblegen human arrays in order to calculate the thermodynamic parameters and model choice [36]. Differences in the probe sequence seems to explain the specific variations of microarray signal intensities as the melting temperature is different for each probe set. In our experiments the 25-mers arrays were hybridized at 38°C, and the 60-mers arrays at 42°C, conforming to Nimblegen recommendations.

**Figure 2:**
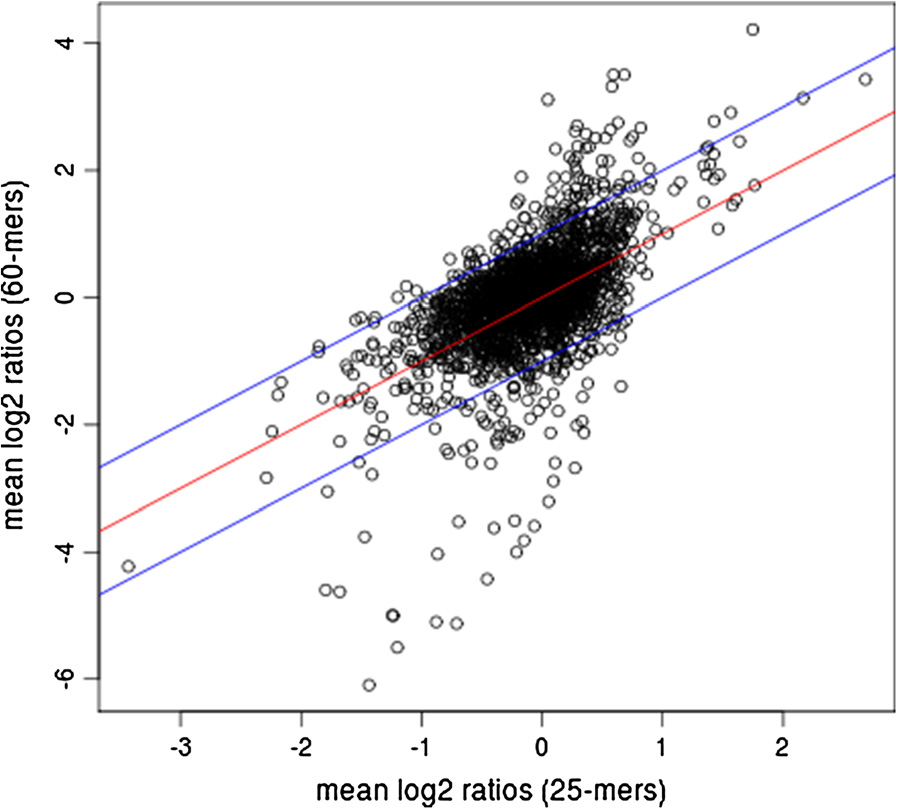
**The inter-array type’s correlation of expression profiles.** Scatter plot was realized using means of log2 ratio of probe intensities in all replicates and shows a strong correlation between the two array types.

Inter-array comparison of expression profiles (Figure 2) showed that a strong correlation exists between the two arrays. Only a few exceptions were detected for up-regulated profiles (log2 ratio >1) on 60-mers arrays and for down-regulated profiles (log2 ratio <−1) on 25-mers arrays.

### Comparison of the accuracy of expression measurements

**Figure 3:**
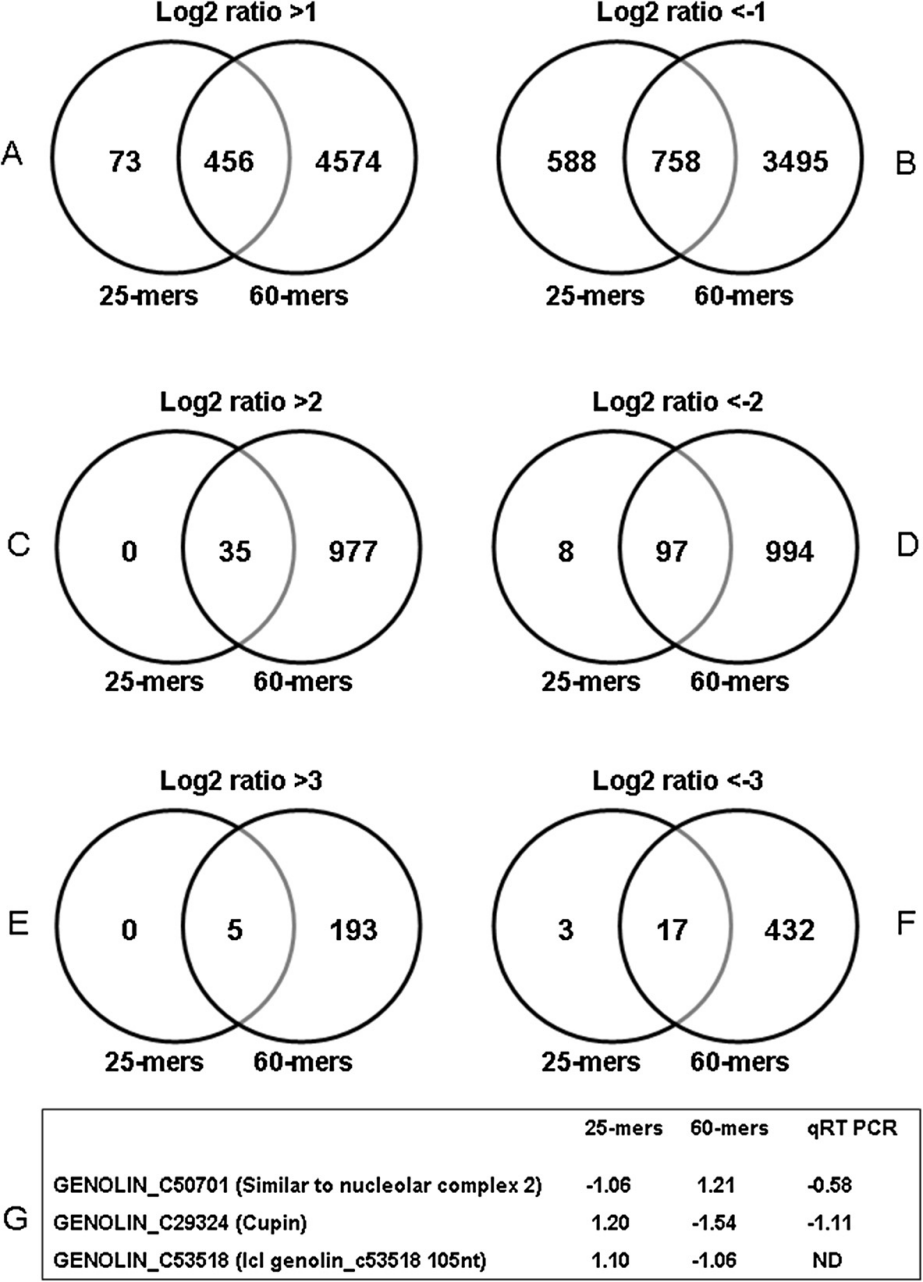
**Venn diagrams representing the number of targets showing significant differences in expression values between each pair-wise combination of inner *vs. *outer tissues represented for three different thresholds: -1< log2 ratio >1 (A and B), -2< log2 ratio >2 (C and D) and −3< log2 ratio >3 (E and F). G**: unigenes showing significantly opposed expression values on the two arrays.

Accuracy is defined as the degree of conformity of the measured quantity to its actual (true) value [28,37]. To evaluate this parameter we used: 1) the number of targets showing differences in expression values between each pair-wise combination of replica slides, and 2) the concordance between relative expression values obtained on arrays with those obtained by qRT-PCR for a subset of 9 genes. The number of targets showing significant differences in expression values between each pair-wise combination of inner *vs.* outer tissues (Figure 3) are given for three different thresholds: -1< log2 ratio >1 (Figures 3A and 3B), -2< log 2ratio >2 (Figures 3C and 3D), and −3< log2 ratio >3 (Figures 3E and 3F). Significantly expressed targets were detected by both arrays at all threshold values used.

The 60-mers arrays showed a much greater sensibility in significant target expression and the number of up- or down-regulated targets was between 4x and 39x more important than with 25-mers arrays depending upon the threshold value used (Additional file 2). This sensibility of detection could be related to the higher intensities of signals in 60-mers arrays (Figure 1A) and the hybridization efficiency that is sequence-dependent (see Comparison of the precision of expression measurements).

**Figure 4:**
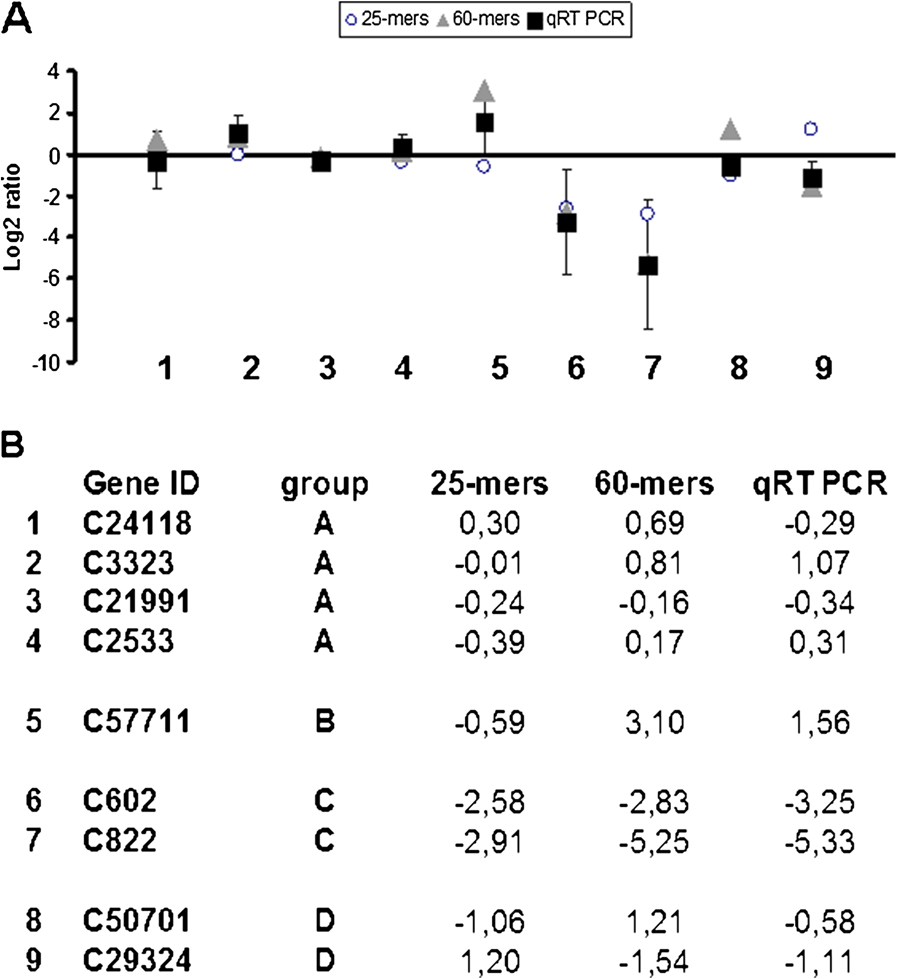
**Correlations of 25-mers and 60-mers array data with sample-matched qRT-PCR data.** Standard deviation of qRT-PCR data were represented as bars. Tested genes were: showing **A**) no significant (1<log2 ratio>−1) expression values on both arrays (C24118, C3323, C21991, C2533), **B**) significant expression value (log2 ratio>1 or log2 ratio<−1) on one array, but not the other (C57711), C) significant expression values on both arrays (C602, C822) and D) significant but opposed expression values (C50701, C29324). For 5 out of the 9 tested genes, qRT-PCR determined expression values were not significantly different from those determined by both flax arrays. For three other genes, qRT-PCR expression values were significantly different from 25-mers data, but not from 60-mers data. The qRT-PCR expression value of one gene was significantly different from 60-mers data but not from 25-mers data.

The 25-mers arrays seemed to produce accurate measurements as a high number of identified targets were confirmed by 60-mers arrays (between 56 and 100% of significant expression values obtained in 25-mers arrays were detected in 60-mers arrays at the same threshold). Targets that were specifically detected using the 25- or 60-mers arrays generally presented relatively similar log2 ratio values even though they did not necessarily occur within the same threshold range. Only three targets (out of the 46,589 unigenes targeted) showed discordant expression values being significantly up-/down-regulated in one array type as compared to the other (Figure 3G). In an attempt to understand this discordance we measured the expression levels of 2 of these genes (C29324: up-regulated on the 25-mers array and down-regulated on the 60-mers array, and C50701: down-regulated on the 25-mers array and up-regulated on the 60-mers array) by qRT-PCR. Our results (Figures 3G and 4) show that qRT-PCR measurements indicate that C50701 is under-expressed in stem inner tissues in agreement with the 25-mers value but in disagreement with the 60-mers value. In contrast the qRT-PCR results indicated that C29324 was up-regulated in inner stem tissues in agreement with the 60-mers results, but not the 25-mers results. One possible explanation for the observed differences could be the existence of alternative splicing variants. Comparison of splicing predictions between genomic (http://www.phytozome.org) and EST (http://urgi.versailles.inra.fr/Species/Flax/Download-sequences) databases suggest that splice variants could exist for C50701. Nevertheless alignment of 25-mer, 60-mer probes and qRT-PCR primers (Additional file 3) showed that none would be capable of distinguishing the different potential splice variants. Further investigation using microarrays specifically designed as tiling or splice junction arrays could provide further information. Comparison of the genomic sequence, probes and primers for the C29324 unigene (Additional file 3) also provided a possible explanation for the observed discordance between 25-mers and 60-mers values. The genomic sequence (*Lus*10011816) and the C29324 unigene show good alignment in the central region of the unigene but are not aligned at both extremities suggesting that the C29324 contig is not correctly assembled. Both 60-mers probes and qRT-PCR primers target the central region (correct) of the gene/transcript whereas the 25-mers target the extremities that are problematic. In conclusion, only 3 out of the 46,589 unigenes (0.0064%) targeted by the 25-mers-arrays and the 60-mers arrays showed discordant significant expressions thereby confirming the overall conformity of probe design for the 2 array platforms. Verification by qRT-PCR and analyses of sequence data for 2 unigenes showing discordant expression values indicated possible explanations for the observed contradictions.

We have previously evaluated the accuracy of the 25-mers platform by qRT-PCR cross-validation using 9 genes [24]. We therefore adopted the same approach to validate the 60-mer platform using a subset of 9 genes showing i) no significant (1<log2 ratio>−1) expression values on both arrays (C24118, C3323, C21991, C2533), ii) significant expression value (log2 ratio>1 or log2 ratio<−1) on one array, but not the other (C57711), iii) significant expression values on both arrays (C602, C822) and iv) significant but opposed expression values (C50701, C29324) (Figure 4). For 5 out of the 9 tested genes (C24118, C3323, C21991, C602, and C2533), qRT-PCR determined expression values were not significantly different from those determined by both flax arrays. For three other genes (C822, C57711, C29324), qRT-PCR expression values were significantly different from 25-mers data, but not from 60-mers data suggesting that the 60-mers array performs better than the 25-mers. The qRT-PCR expression value of only one gene (C50701) was significantly different from 60-mers data but not from 25-mers data. Although examination of genomic and transcript data suggested that different splice variants might exist for this gene as indicated above, neither the 25-mers, nor the 60-mers probes would distinguish the different forms and it is therefore difficult to explain why the 25-mers apparently give a more accurate measure of expression levels for this unigene. The design and use of different qRT-PCR primers and further sequence analyses would enable to clarify this point.

**Figure 5:**
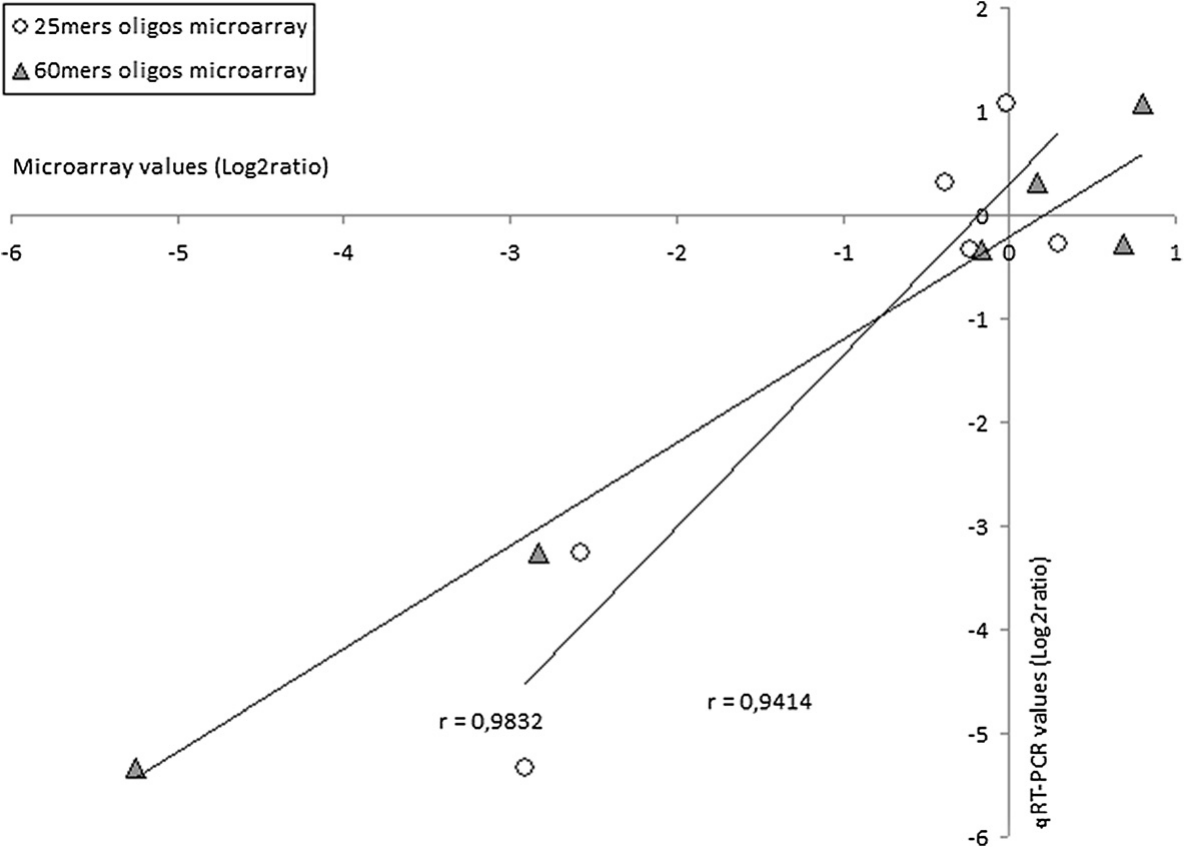
**Correlations between qRT-PCR and microarray results.** Statistically significant correlations were obtained for both 60-mers arrays (r = 0.9832), and 25-mers arrays (r = 0.9414).

The correlation coefficient was calculated separately between the 60-mers results and the qRT-PCR results for 6 selected genes. We deliberately excluded the unigenes C50701 and C29324 that gave discordant results between the platforms and/or the qRT-PCR data, probably resulting from assembly problems as indicated above. We also decided to exclude the unigene C57711 because of the discordant 25-mers and 60-mers values, but also because we were unable to identify the corresponding genomic sequence. The obtained value (r = 0.9832) indicated a highly statistically significant correlation (Figure 5). A similar calculation for the 25-mers platform (Figure 5) also gave a highly significant (but lower) r value (0.9414). Taken together these data indicate a good accuracy for both array types and are in agreement with other similar studies demonstrating that experimental errors were not a significant source of unwanted variability in expression profiling obtained by Affymetrix U74Av2 arrays transcriptome experiments [1] or custom made microarrays [3].

### Differential gene expression in flax tissues

**Figure 6:**
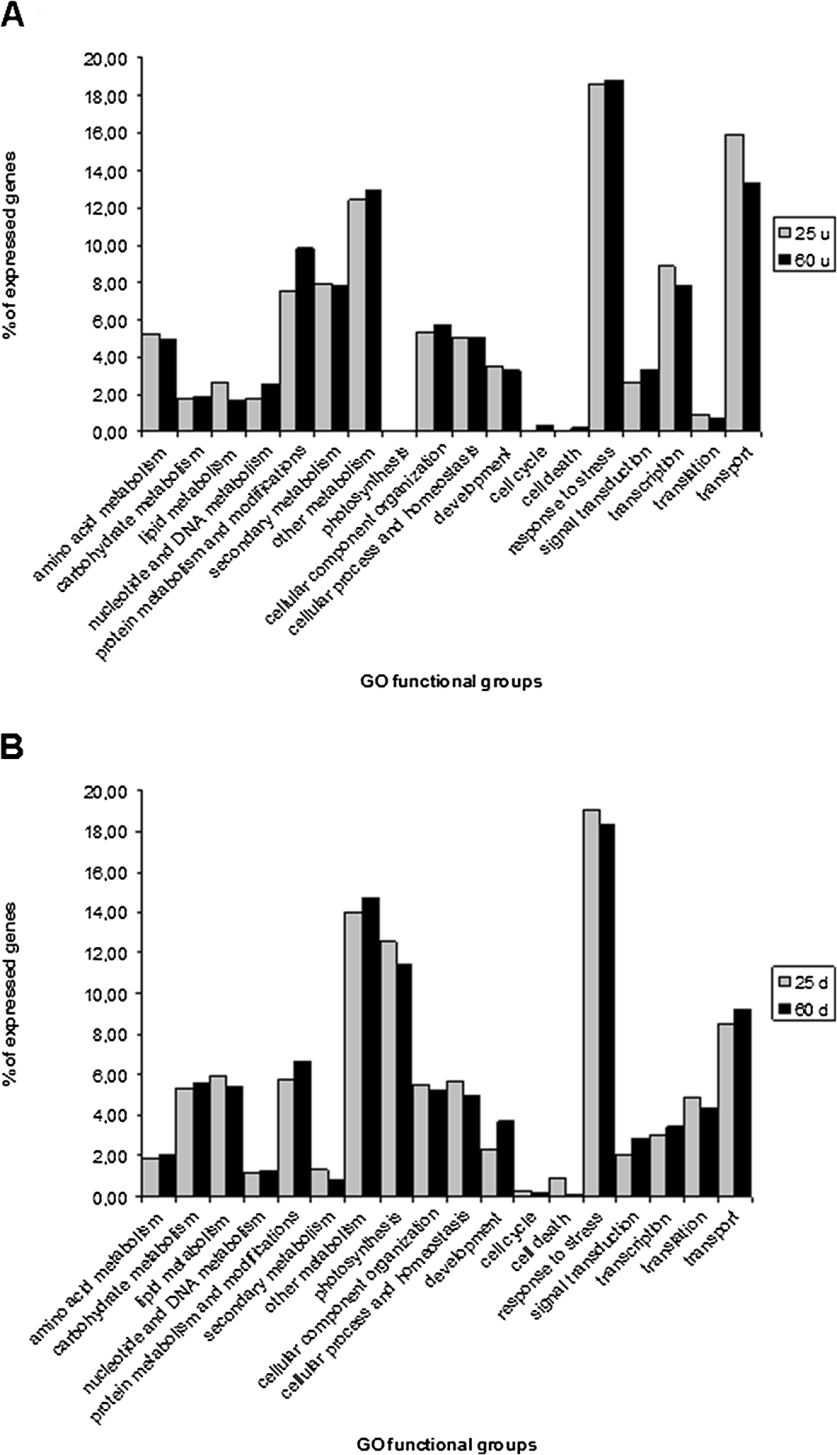
**GO functional classification of up-regulated genes (log2 ratio>1) (A) and down-regulated genes (log2 ratio<−1) (B) in inner *vs. *outer tissues identified by 25- and 60-mers flax Nimblegen arrays.**

Our results showed that the 60-mers array detected a higher number of unigenes differentially expressed between the two flax samples and was therefore more sensitive than the 25-mers array. However, we wondered whether the increased sensitivity also represented an augmentation in the biologically-relevant information. As a first step to answering this question we functionally classified genes showing significant differential expression on the two arrays using GO (Gene Ontology in biological process category) annotations based on blast results and GOA and TAIR gene cross-referenced files [24,26]. Functional categories of up- and down-regulated genes in inner *vs.* outer tissues at −1<log2 ratio<1 in the two array types are represented in Figure 6 and Additional file 2. Even if the total number of significantly expressed genes is very different in the two array types (number up-regulated genes: 529 for 25-mers, 5,030 for 60-mers; number down-regulated genes: 1,346 for 25-mers; 4,253 for 60-mers), the percentages of annotated genes involved in different functional groups are very similar for the 2 arrays. For example, 7.96%, (9 genes) and 7.84%, (43 genes) of all genes significantly more expressed in stem inner tissues were assigned to the class ‘secondary metabolites’ in the 25-mers and 60-mers data sets, respectively. Similarly, 18.58%, (102 genes) and 18.8% (277 genes) were assigned to the class ‘response to stress’, and transport 15.93%, (18 genes) and 13.32%, 87 genes) were assigned to the class ‘transport’ in the 25-mers and 60-mers data sets, respectively. The high similarity between functional class percentage values was also observed for genes showing a significant higher expression in stem outer tissues. For example, 12.56% (86 genes) and 11.41% (170 genes) were assigned to the class ‘photosynthesis’, and 19.05% (132 genes) and 18.35% (275 genes) were assigned to the class ‘response to stress’ in the 25-mers and 60-mers data sets, respectively. Generally, these observations are in close agreement with the known physiological roles of these two different tissues [26,38,39] and confirm the biological consistency of data reported by both array types. Taken together, these observations suggest that both arrays are able to provide a biologically-coherent global view of the flax stem transcriptome.

**Figure 7:**
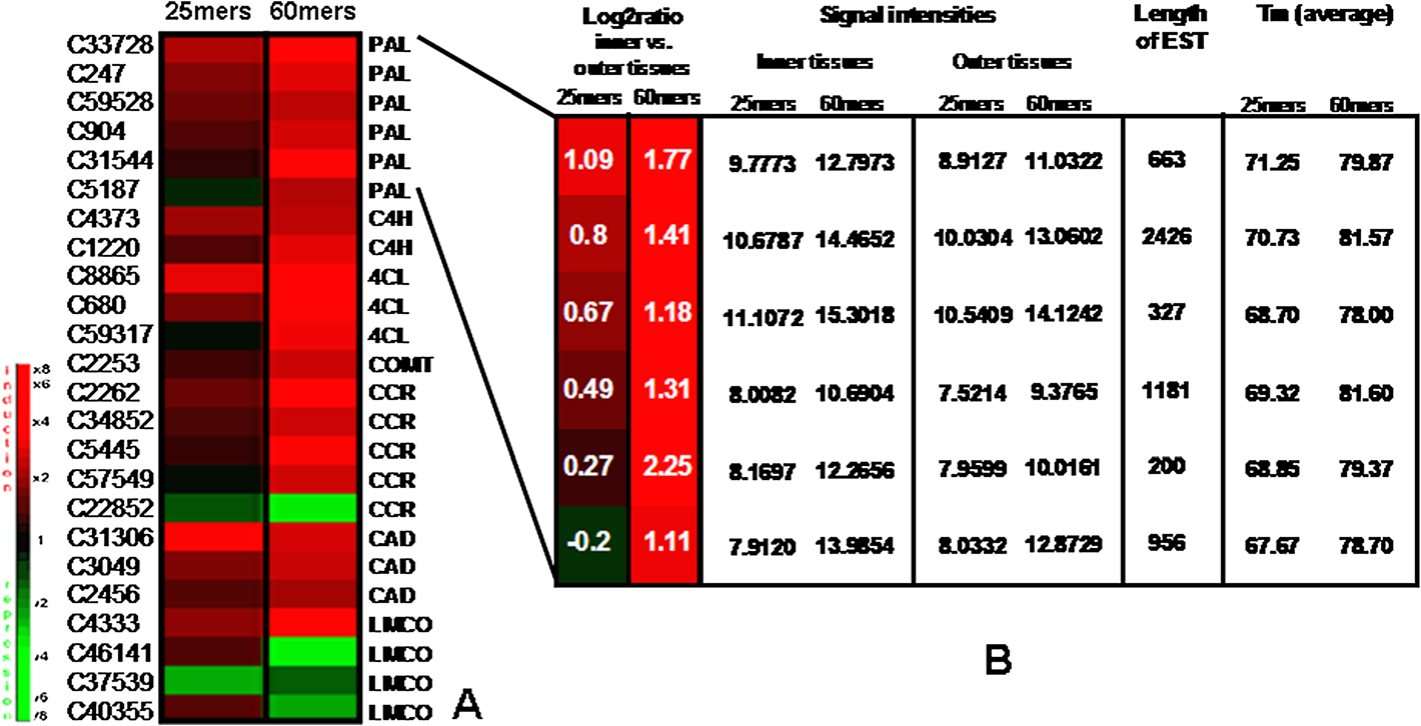
**A: Cluster representing expression profiles of unigenes involved in phenylpropanoid metabolism.** First and second columns represent log2 ratio of inner *vs.* outer tissues in 25- and 60-mers arrays respectively. **B**: Cluster representing expression profiles of *PAL* (*Phenylalanine Ammonia Lyase*) unigenes associated with signal intensities, unigene length and average melting temperartures (Tm) for probes.

In order to better assess whether the increase in the number of significantly expressed genes detected by the 60-mers arrays as compared to the 25-mers arrays represented biologically relevant information we decided to focus on genes encoding enzymes responsible for the biosynthesis of lignin monomers (monolignols) and/or their oxidation (laccases). Our results (Figure 7A) show that the 25-mers array detected 3 significantly expressed unigenes corresponding to 3 multigenes families (*Phenylalanine Ammonia Lyase*: *PAL*, *4-Coumarate Ligase*: *4CL*, *Cinnamyl Alcohol Dehydrogenase*: *CAD*) encoding enzymes involved in monolignol biosynthesis. When the 60-mers array was used additional significantly expressed unigenes corresponding to each of these 3 multigene families were detected (5 *PAL* unigenes, 2 *4CL* unigenes and 2 *CAD* unigenes). Nevertheless, the significantly expressed unigene detected by the 25-mers array also corresponded to the most significantly expressed unigene detected by the 60-mers array. In addition, the 60-mers array, but not the 25-mers array, also detected significantly expressed unigenes corresponding to 3 further multigene families encoding enzymes involved in monolignol biosynthesis (*Cinnamate 4-Hydroxylase*: *C4H*, *Caffeic Acid O-methyltransferase: COMT*, *Cinnamoyl CoenzymeA Reducatse*: *CCR*). Although the *C4H* unigene expression level determined by the 25-mer array was just below the cut-off value (0.98), the *COMT* unigene expression level was considerably inferior (0.37). Similarly all *CCR* unigene expression levels determined by the 25-mers array were well below the threshold value. Similar observations could be made for those unigenes encoding enzymes (laccases) potentially involved in the oxidative polymerisation step of the lignification process. Interestingly however, one laccase unigene (C37539) showed a significant expression with the 25-mers array but not the 60-mers array.

In order to understand the possible reason for the higher sensitivity of the 60-mers arrays, we focused on *PAL* unigenes (Figure 7B) and examined signal intensities, unigene lengths, probe Tm, as well as the probe position and coverage of the EST (Additional file 4). Signal intensities were consistently higher on 60-mers arrays, presumably since the probe Tm average was higher, resulting in lower background. No relation was found between unigene length and array sensitivity. Both arrays covered similar unigene region lengths, generally the 60-mers probes cover 240 bp (4 duplicate probes per unigene) and the 25-mers probes cover 200 pb (8 25-bp probes per unigene) (Additional file 4).

All these observations support the hypothesis that hybridization efficiency depends on probe thermodynamic parameters as previously suggested [33]. Similar results were found with the Agilent platform [40] when 25- and 60-mers arrays were compared. Agilent 60-mers arrays tended to have higher sensitivity, with an average lower detection limit as compared to 25-mers. In contrast, reproducibility of log2 ratio values, system noise and accuracies of log2 ratio determination were comparable between these two microarray types. Similarly, the overall biological information obtained with these 2 arrays was similar in agreement with our observations in flax stem tissues.

## Conclusions

Our study compared two different flax Nimblegen high density microarray platforms based on a short- and long-oligonucleotide design. Our results showed that both array types yielded reproducible, accurate and comparable data that are coherent for expression measurements and identification of differentially expressed genes. Nevertheless, we found that the 60-mers arrays gave higher hybridization efficiencies and therefore were more sensitive allowing the detection of a higher number of unigenes involved in the same biological process and/or belonging to the same multigene family. The two flax arrays provide a good resolution of expressed functions; however the 60-mers arrays are more sensitive and provide a more in-depth coverage of candidate genes potentially involved in different biological processes.

## Methods

### Plant material

*Linum usitatissimum* L. (cv. Barbara) plants were grown in a growth chamber (light/night cycles 16h (22°C)/8h (19°C), 50% humidity and light intensity of 400 μE s-1 m-2) and harvested after nine weeks of grown. The outer fiber-bearing tissues were peeled off and inner tissues (xylem) from a 15 cm long stem section were cut into short fragments before both tissues were frozen in liquid nitrogen as previously described [24,26].

### RNA extraction

Total RNA was isolated from pooled flax inner- and outer-stems using the NucleoSpin® RNA Plant kit (Macherey-Nagel) following manufacturer’s guidelines. To obtain sufficient amount of RNA for microarray analysis (10 μg), a minimum of three extractions with up to 150 mg of fresh tissue were necessary for each sample. To eliminate DNA contamination, on column treatment was done using the RNAse-free DNAse included in the kit. RNA integrity and concentration were evaluated with RNA StdSens Chips using the ExperionTM automated eletrophoresis system (Bio-Rad). For each sample, the three RNA extracts were pooled and final concentrations were adjusted to 1 μg/μL.

### Microarray design and oligo synthesis

Two types (25-mers, 60-mers) of high-density flax microarrays based on the Nimblegen 385K design format (Nimblegen Systems, Inc., Madison, WI, USA) each containing a total of 384,168 oligonucleotides were designed. The 25-mers array utilized 8 distinct, 25 bp-long oligos for each of the 48,021 contigs and the 60-mers array utilized 4 duplicate, 60 bp-long oligonucleotides for 46,589 contigs. Microarray contigs were selected from a collection of 59,000 contigs obtained by assembling the GS FLX sequences [24]. The 46,589 contigs targeted by the 60-mers array were also targeted by the 25-mers array allowing direct comparison between the two designs.

### cDNA synthesis, labeling and hybridization

Double-stranded cDNA (ds-cDNA) was synthesized from 10 μg of total RNA using an Invitrogen SuperScript ds-cDNA synthesis kit in the presence of 250 ng random hexamer primers. ds-cDNA was cleaned and labeled in accordance with the Nimblegen Gene Expression Analysis protocol (Nimblegen Systems, Inc., Madison, WI, USA). Briefly, ds-cDNA was incubated with 4 μg RNase A (Promega) at 37°C for 10 min and cleaned using phenol:chloroform:isoamyl alcohol, followed by ice-cold absolute ethanol precipitation. For Cy3 labeling of cDNA, the Nimblegen One-Color DNA labeling kit was used according to the manufacturer’s guideline detailed in the Gene Expression Analysis protocol (Nimblegen Systems, Inc., Madison, WI, USA). One μg ds-cDNA was incubated for 10 min at 98°C with 2 OD of Cy3-9mer primer. Then, 100 pmol of deoxynucleoside triphosphates and 100U of the Klenow fragment (New England Biolabs, Ipswich, MA, USA) were added and the mix incubated at 37°C for 2h30. The reaction was stopped by adding 0.1 volume of 0.5 M EDTA, and the labeled ds-cDNA was purified by isopropanol/ethanol precipitation. Microarrays were hybridized at 38°C (25-mers arrays) and at 42°C (60-mers arrays) during 16 to 18h with 6μg of Cy3 labelled ds-cDNA in Nimblegen hybridization buffer/hybridization component A in a hybridization chamber (Hybridization System - Nimblegen Systems, Inc., Madison, WI, USA). Following hybridization, washing was performed using the Nimblegen Wash Buffer kit (Nimblegen Systems, Inc., Madison, WI, USA).

### Data analysis

Slides were scanned at 5 μm/pixel resolution using an Axon GenePix 4000B scanner (Molecular Devices Corporation, Sunnyvale, CA, USA) piloted by GenePix Pro 6.0 software (Axon). Scanned images (TIFF format) were then imported into NimbleScan software (Nimblegen Systems, Inc., Madison, WI, USA) for grid alignment and expression data analyses. Expression data were normalized through quantile normalization [41] and the Robust Multichip Average (RMA) algorithm [42] included in the NimbleScan software. Identification of genes displaying a change in expression over repetitions was accomplished with a script utilizing library functions in R with a false discovery rate (FDR) of less than 5%. The SAM [43] was used to identify differentially expressed genes over different conditions. Analysis was completed with the Tree-view clustering program [44]. Functional annotation of differentially-expressed genes was based on Gene Ontology (http://www.geneontology.org/). All the microarray data have been submitted to the Gene Expression Omnibus (GEO) database [45] with the accession number is GSE37980.

### Quantitative reverse transcriptase-PCR (qRT-PCR) analysis

For qRT-PCR analyses, 1 μg of total RNA was reverse-transcribed to single stranded cDNA using the IScript cDNA synthesis kit (Bio-Rad) according to the manufacturer’s instructions. The qRT-PCRs were carried out in 96-wells plates with a MyIQ real time PCR detection system (Bio-Rad) using iQSYBR Green PCR Kit (Bio-Rad) in a reaction volume of 20 μL (5 μL diluted cDNAs, 10 μL of 2× SYBR Green mix and primer pairs at 0.4 μM). Aliquots from the same cDNA solutions were used with all primer sets in each experiment. All PCR reactions were performed under the following conditions: 95°C for 15 min, 40 cycles of 10 s at 95°C and 30 s at 60°C. For each primer pair, a melting curve was generated in order to confirm the specificity of the amplification. The primer sequences used for all target genes are presented in Additional file 5.

Each experiment was repeated on three biological replicates, each one represented by three technical repetitions. PCR reactions on samples lacking the cDNA template or the reverse transcriptase during the cDNA synthesis were also performed as negative controls for each primer pair. The efficiency (E) value of each reaction was between 0.85 and 1.17 with R2 values higher than 0.99.

Data were analysed using Bio-Rad iQ5 software. For each primer pair, a melting curve was generated in order to confirm the specificity of the amplification. The PCR efficiencies (E) for each reaction were between 0.85 and 1.17 with R^2^ values higher than 0.99. The expression of each gene was normalized by using 2 reference genes, *ETIF1* and *ETIF4F*, shown to be expressed in a stable manner in flax stem tissues [46].

## Abbreviations

NGS: Next generation sequencing; RMA: Robust multi-array average; CV: Coefficient of variation; SD: Standard deviation; qRT-PCR: quantitative Real Time Polymerase Chain Reaction; GO: Gene ontology; GOA: Gene ontology annotation; TAIR: The arabidopsis information resource; PAL: Phenylalanine ammonia lyase; 4CL: 4-Coumarate ligase; CAD: Cinnamyl alcohol dehydrogenase; C4H: Cinnamate 4-Hydroxylase; COMT: Caffeic acid O-methyltransferase; CCR: Cinnamoyl coenzymeA reducatse; EST: Expressed sequence tag; GEO: Gene expression omnibus.

## Competing interests

The authors declare that they have no competing interests. No funding either now or in the future, no stocks or shares was or will be perceived, no interest or financing is known from an organisation for this manuscript. No patents relating to the content of the manuscript are known, and no non-financial competing interests are to declare in relation to this manuscript.

## Authors’ contributions

SF and RH realized the plant cultures, RNA extractions, and transcriptomic analyses. MC and GN performed the qRT-PCR analyses, SG executed the biocomputing analyses of the results, NR and BT contributed to the microarrays design and construction, SH participated in the writing and global interpretations of paper, AL organized the various strategies of analyses and interpretation and drafted the paper. All authors read and approved the final manuscript.

## Authors’ information

SF is a researcher in post doctorat position; MC is a PhD student; SG is bioinformatics ingeneer; RH is researcher; GN is assistant professor in charge of qRT-PCR platform; NR is the head of Biogemma’s transcriptomics group; BT is professor; SH is professor and the Plant FibreTeam leader; AL is assistant professor in charge of transcriptomic platform.

## Supplementary Material

Additional file 1 Representative image of flax stem tissues. Click here for file

Additional file 2 **Average of differentially expressed genes in inner *vs.* outer tissues in 25 and 60-mers arrays.** Click here for file

Additional file 3 Alignment of microarray probes, qRT-PCR primers and unigene sequences for C50701 and C29324. Click here for file

Additional file 4 Coverage of phenylalanine ammonia lyase ESTs by 25-mers and 60-mers probes. Click here for file

Additional file 5 Primer design for qRT-PCR. Click here for file
