# Supplementary material for: Intra-platform comparison of 25-mer and 60-mer oligonucleotide Nimblegen DNA microarrays
Source: BMC Res Notes. 2013 Feb 4;6:43. doi: 10.1186/1756-0500-6-43 (PMC3608165; doi:10.1186/1756-0500-6-43)

Additional file 1. Representative image of samples and associated marker genes

Stem cross-sections of flax stem shows inner tissues (I) mostly represented by xylem (X), and outer tissues (O) mostly represented by cortical parenchyma (C), bast fibers (B), and phloem (P). Cross-sections are stained with TBO (toluidine blue O, 0.1% w/ml). Bar = 20 µm.

Preliminary examination of microarray data (both platforms) and qRT-PCR data indicated that marker genes characteristic of inner tissues (e.g. genes encoding enzymes involved in monolignol biosynthesis and polymerization) and outer tissues (e.g. genes involved in photosyntheisis) were significantly up-regulated in the appropriate tissues thereby confirming sample identity and absence of contamination.

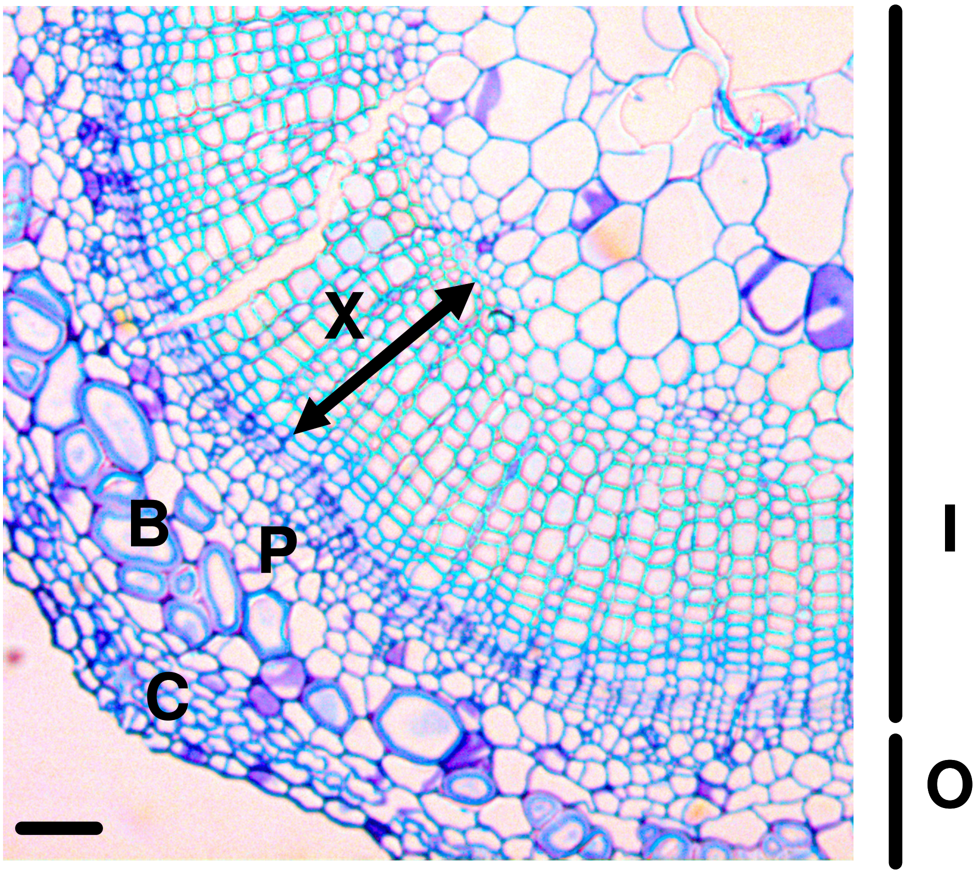

Supplement: Additional file 1 — Representative image of flax stem tissues. [file 1756-0500-6-43-S1.pdf]
