# Supplementary material for: Intra-platform comparison of 25-mer and 60-mer oligonucleotide Nimblegen DNA microarrays
Source: BMC Res Notes. 2013 Feb 4;6:43. doi: 10.1186/1756-0500-6-43 (PMC3608165; doi:10.1186/1756-0500-6-43)
Supplement: Additional file 3 — Alignment of microarray probes, qRT-PCR primers and unigene sequences for C50701 and C29324. [file 1756-0500-6-43-S3.pdf]

Additional file 3. Alignment of microarray probes, qRT-PCR primers and unigene sequences for C50701 and C29324.

Alignment of 25-mer/60-mer probes to unigene sequences, and qRT-PCR amplified regions are represented in blue. Genomic sequences and alignements of genolin unigenes to CDS sequences (http://www.phytozome.net/search.php) are also represented.

genolin_c50701 coverage by 25-mers probes

TATCACCAGTAGCAACAAATTCAATTCTAACCAACYRKSTWYKGMWSKAGTTTACGTGCTCTTTCAGGGGGAGGCTTGCTGT
CGTCAGTTGTTTTTGCTTCTTCTGTGAACGCCAACCATGGGAAAGCTAGGAAAGAATGCGAGRAAGTTCGCMAAGAAGAATC
TTCAGTCAGTAATGAAAAGGCAGAGGAAGATAAAGTCCGTTTTCAAGAAAAGAGCTACRAAGGAAAGGGGGGCGTGGAGGT
GGWAATCATGRARAGGAGATAA

genolin_c50701 coverage by 60-mers probes

TATCACCAGTAGCAACAAATTCAATTCTAACCAACYRKSTWYKGMWSKAGTTTACGTGCTCTTTCAGGGGGAGGCTTGCTGT
CGTCAGTTGTTTTTGCTTCTTCTGTGAACGCCAACCATGGGAAAGCTAGGAAAGAATGCGAGRAAGTTCGCMAAGAAGAATC
TTCAGTCAGTAATGAAAAGGCAGAGGAAGATAAAGTCCGTTTTCAAGAAAAGAGCTACRAAGGAAAGGGGGGCGTGGAGGT
GGWAATCATGRARAGGAGATAA

genolin_c50701 qRT PCR amplified region

TATCACCAGTAGCAACAAATTCAATTCTAACCAACYRKSTWYKGMWSKAGTTTACGTGCTCTTTCAGGGGGAGGCTTGCTGT
CGTCAGTTGTTTTTGCTTCTTCTGTGAACGCCAACCATGGGAAAGCTAGGAAAGAATGCGAGRAAGTTCGCMAAGAAGAATC
TTCAGTCAGTAATGAAAAGGCAGAGGAAGATAAAGTCCGTTTTCAAGAAAAGAGCTACRAAGGAAAGGGGGGCGTGGAGGT
GGWAATCATGRARAGGAGATAA

genomic sequence Lus10041417

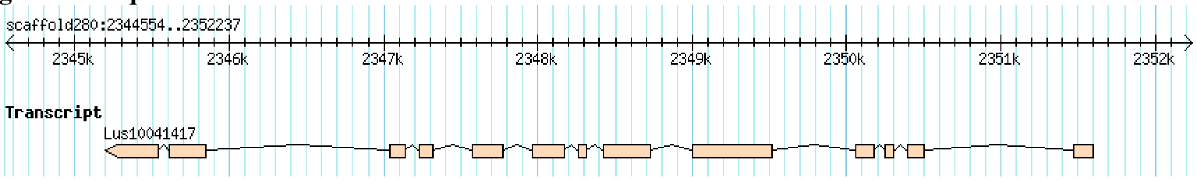

alignment of CDS sequence Lus10041417 (Query) and genolin_c50701 (Subject)

```
Score =  196 bits (102), Expect = 3e-47
Identities = 105/108 (97%)
Strand = Plus / Plus
```

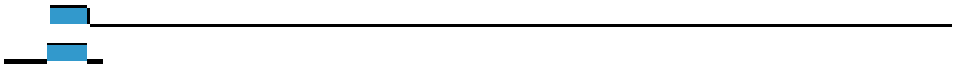

```
Query: 1   atgggaaagctaggaaagaatgcgagaaagttcgcaaagaagaatcttcagtcagtaatg 60
           |||||||||||||||||||||||||| |||||||| |||||||||||||||||||||||||
Sbjct: 119 atgggaaagctaggaaagaatgcgagraagttcgcmaagaagaatcttcagtcagtaatg 178


Query: 61  aaaaggcagaggaagataaagtccgttttcaagaaaagagctacgaag 108
           |||||||||||||||||||||||||||||||||||||||||||||| |||
Sbjct: 179 aaaaggcagaggaagataaagtccgttttcaagaaaagagctacraag 226
```

genolin_c29324 coverage by 25-mers probes

TTTTTTCGGGGGGTTTTTTCGTTTTTTTTCCTTTTTACGTTTTATTTTAGGGKCGTATTCCGAGGGATGTGCTGGCTAACGCGTTC
GGAGTGTCGAGGGAGGAGGCCCAGAGGATCAAGTTTAGCCGGAGGGAGGTTTCGCTCTTCAGCCCTGGTAACCAGTCTCAGC
AGAGGAGGGACTACTGAGTTTTATGATATAACTAATAAGAAGTAAAATAATGTTTAATAAATAATAAAAGTAATAAAGCTGG
TTGTATTTTCGCGGGACTAAAATGGATGTTTGTATCGTGAGTTCAAATAATGAAATAAAAGAATATAATTTAAAAACTAGT

genolin_c29324 coverage by 60-mers probes

TTTTTTCGGGGGGTTTTTTCGTTTTTTTTCCTTTTTACGTTTTATTTTAGGGKCGTATTCCGAGGGATGTGCTGGCTAACGCGTTC
GGAGTGTCGAGGGAGGAGGCCCAGAGGATCAAGTTTAGCCGGAGGGAGGTTTCGCTCTTCAGCCCTGGTAACCAGTCTCAGC
AGAGGAGGGACTACTGAGTTTTATGATATAACTAATAAGAAGTAAAATAATGTTTAATAAATAATAAAAGTAATAAAGCTGG
TTGTATTTTCGCGGGACTAAAATGGATGTTTGTATCGTGAGTTCAAATAATGAAATAAAAGAATATAATTTAAAAACTAGT

genolin_c29324 qRT PCR amplified region

TTTTTTCGGGGGGTTTTTTCGTTTTTTTTCCTTTTTACGTTTTATTTTAGGGKCGTATTCGAGGGATGTGCTGGCTAACGCGTTC
GGAGTGTCGAGGGAGGAGGCCCAGAGGATCAAGTTTAGCCGGAGGGAGGTTTCGCTCTTCAGCCCTGGTAACCAGTCTCAGC
AGAGGAGGGACTACTGAGTTTTATGATATAACTAATAAGAAGTAAAATAATGTTTAATAAATAATAAAAGTAATAAAGCTGG
TTGTATTTTCGCGGGACTAAAATGGATGTTTGTATCGTGAGTTCAAATAATGAAATAAAAGAATATAATTTAAAAACTAGT

genomic sequence Lus10011816

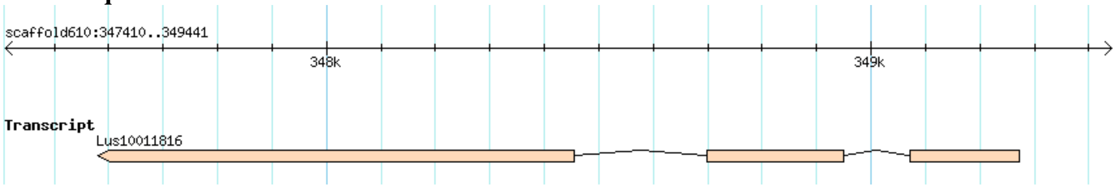

alignment of CDS sequence Lus10011816 (Query) and genolin_c29324 (Subject)

```
Score =  248 bits (129), Expect = 4e-63
Identities = 129/129 (100%)
Strand = Plus / Plus
```

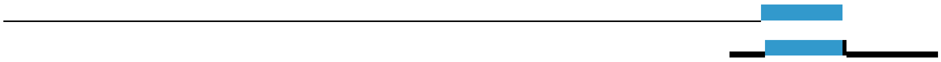

```
Query: 1201 attccgagggatgtgctggctaacgcgttcggagtgtcgagggaggaggcccagaggatc 1260
            ||||||||||||||||||||||||||||||||||||||||||||||||||||||||||||
Sbjct: 56   attccgagggatgtgctggctaacgcgttcggagtgtcgagggaggaggcccagaggatc 115


Query: 1261 aagtttagccggagggaggtttcgctcttcagccctggtaaccagtctcagcagaggagg 1320
            ||||||||||||||||||||||||||||||||||||||||||||||||||||||||||||
Sbjct: 116  aagtttagccggagggaggtttcgctcttcagccctggtaaccagtctcagcagaggagg 175


Query: 1321 gactactga 1329
            |||||||||
Sbjct: 176  gactactga 184
```
