# Supplementary material for: Intra-platform comparison of 25-mer and 60-mer oligonucleotide Nimblegen DNA microarrays
Source: BMC Res Notes. 2013 Feb 4;6:43. doi: 10.1186/1756-0500-6-43 (PMC3608165; doi:10.1186/1756-0500-6-43)
Supplement: Additional file 4 — Coverage of phenylalanine ammonia lyase ESTs by 25-mers and 60-mers probes. [file 1756-0500-6-43-S4.pdf]

## Additional file 4. Coverage of phenylalanine ammonia lyase ESTs by 25-mers and 60-mers probes

**genolin_c33728 coverage by 25-mers probes**
AGGACAGGTACGCTCTCAGGACATCTCCTCAATGGCTCGGCCCCCTGATCGAAGTGATCAGGTCATCGACCAAAATGATTGA
AAGGGAGATCAACTCGGTCAACGACAACCCGTTGATCGATGTCTCGAGAGGGAAGGCAATCCACGGAGGGAACTTCCAGGG
AACACCGATCGGTGTCTCGATGGACAACACTCGTTTGGCAGTTGCTTCAATTGGGAAGCTCATGTTTGCGCAGTTCTCCGAAC
TTGTCAACGACTTTTACAACAACGGTCTGCCATCGAACTTAACAGGTGGACGAAACCCCAGCTTGGACTACGGGTTCAAGGG
GGCTGAAATCGCAATGGCATCCTACTGCTCCGAGCTTCAGTTCCTGGCAAATCCAGTTACAAACCACGTACAGAGCGCTGAAC
AACACAACCAAGATGTCAACTCATTGGGACTGATTTCGGCTAGGAAGACTGCCGAAGCTGTGGACATATTAAAGCTGATGTC
GACAACTTACTTGGTCGCTCTATGCCAAGCGGTCGACTTAAGGTATATCGAGGAGAATCTGAAGCAGACAGTTAAGAACACG
ATCAGCCAAGTAGCGAAGAAAGTACTCCTAACCAACGGGGAGGTGCTCCACCCATCGAGATTCTGCGAGAAGGAGCTATTGA
ATG

**genolin_c33728 coverage by 60-mers probes**
AGGACAGGTACGCTCTCAGGACATCTCCTCAATGGCTCGGCCCCCTGATCGAAGTGATCAGGTCATCGACCAAAATGATTGA
AAGGGAGATCAACTCGGTCAACGACAACCCGTTGATCGATGTCTCGAGAGGGAAGGCAATCCACGGAGGGAACTTCCAGGG
AACACCGATCGGTGTCTCGATGGACAACACTCGTTTGGCAGTTGCTTCAATTGGGAAGCTCATGTTTGCGCAGTTCTCCGAAC
TTGTCAACGACTTTTACAACAACGGTCTGCCATCGAACTTAACAGGTGGACGAAACCCCAGCTTGGACTACGGGTTCAAGGG
GGCTGAAATCGCAATGGCATCCTACTGCTCCGAGCTTCAGTTCCTGGCAAATCCAGTTACAAACCACGTACAGAGCGCTGAAC
AACACAACCAAGATGTCAACTCATTGGGACTGATTTCGGCTAGGAAGACTGCCGAAGCTGTGGACATATTAAAGCTGATGTC
GACAACTTACTTGGTCGCTCTATGCCAAGCGGTCGACTTAAGGTATATCGAGGAGAATCTGAAGCAGACAGTTAAGAACACG
ATCAGCCAAGTAGCGAAGAAAGTACTCCTAACCAACGGGGAGGTGCTCCACCCATCGAGATTCTGCGAGAAGGAGCTATTGA
ATG

**genolin_c247 coverage by 25-mers probes**
CAAAAAAGACAATGGAGCTCTGCAGCAACGAGAACAACAACAATGGTGGTGGGTTTTCCCTCGCTGACCCGTTGAACTGGGG
AATGGCGGCAGAGTCGATGAAAGGGAGCCACTTGGAGGAGGTGAAGCGTATGGTTGAAGAGTCGAGATCCCTGTCGTCAAG
CTTGCCGGGCAGACTCTGTCCATTGCCCAAGTCGTCGCAATTTCCCGCTCCGACGCCTCGGCTGAGCTCGACGAGGAGGCCCG
GCCAAGGGTGAAGGCCAGCAGCGACTGGGTGATGGAGAGCATGAACAAAGGCACGGACAGTTACGGTGTCACGACCGGGTT
CGGAGCTACGTCTCATCGTAGGACTAAGCAGGGTGGAGCCCTTCAGAGGGAGCTCATCAGGTTCTTGAACGCTGGAATATTC
GGCAACGGTACAGAGTCAAACCACACGCTGCCATACTCCGCGACCAGGGCAGCCATGCTGGTCAGGATCAACACCTTGCTCC
AGGGCTATTCAGGAATCCGTTTCGAAATTCTCGAGGCCATCACCAAGCTCCTCAACAACAATGTCACCCCTTGTCTACCTCTC
AGGGGCACCATCACCGCCTCTGGTGACTTGGTCCCTCTATCCTACATTGCAGGCCTGTTAACCGGCCGGCCCAACTCTAAAGC
TGTCGGCCCCAAGGGCGAGTCCCTCGACGCAACACAAGCCTTCCAGGCCGCTGGGATCACCGGTGGATTCTTCGAGTTGCAG
CCAAAGGAAGGTCTGGCGATGGTGAACGGTACTGCAGTCGGTTCAGGAATGGCCTCGATGGTCCTTTTCGACGCCAACATTTT
GGCGGTCATGTCCGAGGTTCTCTCAGCGATTTTCGCTGAAGTTATGCAAGGTAAACCCGAGTTCACAGACCACTTGACGCATA
AGCTGAAGCATCACCCTGGCCAGATCGAAGCTGCAGCGATCATGGAACATATCCTGAAGGGCAGCTCTTACGTCAAGGAAGC
TGAGAAATTGCACGAAATCGATCCGTTACAGAAGCCGAAACAGGACAGATATGCTCTCAGGACGTCTCCTCAATGGCTCGGC
CCGTTGATCGAAGTGATCAGGTCGTCGACGAAAATGATCGAAAGGGAGATTAATTCGGTCAATGACAATCCATTGATCGACG
TCTCGAGAGGGAAGGCGATCCACGGAGGGAACTTCCAGGGAACCCCGATCGGTGTCTCGATGGACAACACTCGTTTGGCCGT
TGCTTCGATTGGGAAGCTCATGTTTGCACAGTTCTCCGAGCTTGTCAACGACTTCTACAACAACGGTCTGCCATCCAACTTAAC
CGGTGGACGAAACCCCAGCTTGGACTATGGGTTCAAGGGGGCTGAAATCGCGATGGCATCCTACTGCTCCGAGCTTCAGTTCC
TGGCAAATCCAGTTACAAACCATGTACAGAGCGCTGAACAACACAACCAGGATGTCAACTCATTGGGCCTGATTTCGGCTAG
GAAGACTGCCGAAGCTGTGGAGATACTAAAGCTGATGTCGACAACTTACTTGGTCGCTCTATGCCAAGCAGTCGATTTAAGGT
ATATCGAGGAGAATCTGAAGCAGACAGTTAAGAACACGATCAGCCAAGTTGCGAAGAAAGTACTCCTGACCAACGGGGAGC
TGCTCCACCCGTCGAGATTCTGCGAGAAGGAGCTCTTGAATGTTGCAGAGAGGGAGTACTTGTTTGCATACGCAGACGATCCT
TGCAGCGCAACCTACCCTCTGATGCAGAAGCTGAGAGGGGTGCTGGTGGAGCACGCGTTGCTGAACGGTGAGAGCGAGAAA
GACTCGAGCACTTCCATCTTCCATAAGATTGCGGTGTTCGAGGAGGAGCTGAAGGCTATCCTGCCGAAAGAAGTTGAGAATG
CAAGGAGCGAGCTGGAGAAAGGGAATCCGGCTATCCCGAACAGGATCAAGGAATGCAGGTCGTATCCGTTGTACAAGTTTGT
GAGGGAGGAAGTTGGTACCAGTTTGCTTACGGGTGAGAAGGTTAAGTCACCAGGTGAGGAATTTGACAAGGTGTTCACTGCT
ATGTGTGAAGGGAAGCTCATTGACCCTTTGTTGGAGTGCCTCAAAGAATGGAATGGTGCTCCTCTTCCCATCTGCTAGAAGAA
CAAATATAAGCATGAATTACCCCGATGTAATCTTTTTGTATTTTCCTGCAAACAAACTATTATTATCTGATGTAAAAGTGAACT
GCTTTTTGGTTGTTATTTCCTTTCTTTACCCATTAATGTGTAAACTATCAGTGTATCTTCTCTAATTTTTGTTGGGAATCCTTTTC
TGTGTACTGGTTTCAAGACAAACGGATGAAATCTGTATACGAAAGGAAATAATTAAGTCTTCTTGTTGCTTGCAATTGGG

**genolin_c247 coverage by 60-mers probes**
CAAAAAAGACAATGGAGCTCTGCAGCAACGAGAACAACAACAATGGTGGTGGGTTTTCCCTCGCTGACCCGTTGAACTGGGG
AATGGCGGCAGAGTCGATGAAAGGGAGCCACTTGGAGGAGGTGAAGCGTATGGTTGAAGAGTCGAGATCCCTGTCGTCAAG
CTTGCCGGGCAGACTCTGTCCATTGCCCAAGTCGTCGCAATTTCCCGCTCCGACGCCTCGGCTGAGCTCGACGAGGAGGCCCG
GCCAAGGGTGAAGGCCAGCAGCGACTGGGTGATGGAGAGCATGAACAAAGGCACGGACAGTTACGGTGTCACGACCGGGTT
CGGAGCTACGTCTCATCGTAGGACTAAGCAGGGTGGAGCCCTTCAGAGGGAGCTCATCAGGTTCTTGAACGCTGGAATATTC
GGCAACGGTACAGAGTCAAACCACACGCTGCCATACTCCGCGACCAGGGCAGCCATGCTGGTCAGGATCAACACCTTGCTCC
AGGGCTATTCAGGAATCCGTTTCGAAATTCTCGAGGCCATCACCAAGCTCCTCAACAACAATGTCACCCCTTGTCTACCTCTC
AGGGGCACCATCACCGCCTCTGGTGACTTGGTCCCTCTATCCTACATTGCAGGCCTGTTAACCGGCCGGCCCAACTCTAAAGC
TGTCGGCCCCAAGGGCGAGTCCCTCGACGCAACACAAGCCTTCCAGGCCGCTGGGATCACCGGTGGATTCTTCGAGTTGCAG
CCAAAGGAAGGTCTGGCGATGGTGAACGGTACTGCAGTCGGTTCAGGAATGGCCTCGATGGTCCTTTTCGACGCCAACATTTT
GGCGGTCATGTCCGAGGTTCTCTCAGCGATTTTCGCTGAAGTTATGCAAGGTAAACCCGAGTTCACAGACCACTTGACGCATA
AGCTGAAGCATCACCCTGGCCAGATCGAAGCTGCAGCGATCATGGAACATATCCTGAAGGGCAGCTCTTACGTCAAGGAAGC
TGAGAAATTGCACGAAATCGATCCGTTACAGAAGCCGAAACAGGACAGATATGCTCTCAGGACGTCTCCTCAATGGCTCGGC
CCGTTGATCGAAGTGATCAGGTCGTCGACGAAAATGATCGAAAGGGAGATTAATTCGGTCAATGACAATCCATTGATCGACG
TCTCGAGAGGGAAGGCGATCCACGGAGGGAACTTCCAGGGAACCCCGATCGGTGTCTCGATGGACAACACTCGTTTGGCCGT
TGCTTCGATTGGGAAGCTCATGTTTGCACAGTTCTCCGAGCTTGTCAACGACTTCTACAACAACGGTCTGCCATCCAACTTAAC
CGGTGGACGAAACCCCAGCTTGGACTATGGGTTCAAGGGGGCTGAAATCGCGATGGCATCCTACTGCTCCGAGCTTCAGTTCC

TGGCAAATCCAGTTACAAACCATGTACAGAGCGCTGAACAACACAACCAGGATGTCAACTCATTGGGCCTGATTTCGGCTAG
GAAGACTGCCGAAGCTGTGGAGATACTAAAGCTGATGTCGACAACTTACTTGGTCGCTCTATGCCAAGCAGTCGATTTAAGGT
ATATCGAGGAGAATCTGAAGCAGACAGTTAAGAACACGATCAGCCAAGTTGCGAAGAAAGTACTCCTGACCAACGGGGAGC
TGCTCCACCCGTCGAGATTCTGCGAGAAGGAGCTCTTGAATGTTGCAGAGAGGGAGTACTTGTTTGCATACGCAGACGATCCT
TGCAGCGCAACCTACCCTCTGATGCAGAAGCTGAGAGGGGTGCTGGTGGAGCACGCGTTGCTGAACGGTGAGAGCGAGAAA
GACTCGAGCACTTCCATCTTCCATAAGATTGCGGTGTTCGAGGAGGAGCTGAAGGCTATCCTGCCGAAAGAAGTTGAGAATG
CAAGGAGCGAGCTGGAGAAAGGGAATCCGGCTATCCCGAACAGGATCAAGGAATGCAGGTCGTATCCGTTGTACAAGTTTGT
GAGGGAGGAAGTTGGTACCAGTTTGCTTACGGGTGAGAAGGTTAAGTCACCAGGTGAGGAATTTGACAAGGTGTTCACTGCT
ATGTGTGAAGGGAAGCTCATTGACCCTTTGTTGGAGTGCCTCAAAGAATGGAATGGTGCTCCTCTTCCCATCTGCTAGAAGAA
CAAATATAAGCATGAATTACCCCGATGTAATCTTTTTGTATTTCCTGCAAACAAACTATTATTATCTGATGTAAAAGTGAACT
GCTTTTTGGTTGTTATTTCCTTTCTTTACCCATTAATGTGTAAACTATCAGTGTATCTTCTCTAATTTTTGTTGGGAATCCTTTTC
TGTGTACTGGTTTCAAGACAAACGGATGAAATCTGTATACGAAAGGAAATAATTAAGTCTTCTTGTTGCTTGCAATTGGG

**genolin_c59528 coverage by 25-mers probes**
TGCGAGGAGCGAGCTAGAGAATGGGAACCCGGCTATTCCGAACAGGATTAAAGAGTGCAGGTCGTATCCGTTGTACAAGTTT
GTGAGGGAGGAAGTTGGTACCGGTTTGCTGACGGGTGAGAAGGTTAAGTCACCTGGTGAGGAATTTGACAAGGTGTTCACTG
CTATGTGTGAAGGGAAGTTGATTGATCCTTTGTTGGAGTGCCTCAAAGAATGGAATGGTGCTCCTCTTCCTATCTGCTAGAAC
AAGACTTATAAGCATGCATTGCTCTGATGTAATCTTTTGTATTTCCTGCAAACAAACTATTATTATCTGATGTAAAAGT

**genolin_c59528 coverage by 60-mers probes**
TGCGAGGAGCGAGCTAGAGAATGGGAACCCGGCTATTCCGAACAGGATTAAAGAGTGCAGGTCGTATCCGTTGTACAAGTTT
GTGAGGGAGGAAGTTGGTACCGGTTTGCTGACGGGTGAGAAGGTTAAGTCACCTGGTGAGGAATTTGACAAGGTGTTCACTG
CTATGTGTGAAGGGAAGTTGATTGATCCTTTGTTGGAGTGCCTCAAAGAATGGAATGGTGCTCCTCTTCCTATCTGCTAGAAC
AAGACTTATAAGCATGCATTGCTCTGATGTAATCTTTTGTATTTCCTGCAAACAAACTATTATTATCTGATGTAAAAGT

**genolin_c904 coverage by 25-mers probes**
CCTTTAGCAGATTGGGATAGGGGCACCATTCCATTCATGGTGGCAACTTCCAGGGAACTCCCMTYGGRGTGTCGATGGACAA
TGTCCGGTTGGCGATTGCTTCGATCGGGAAGTTAATGTTCGCTCAGTTCAGCGAGCTAGTCAATGATTTCTACAACAATGGAC
TGCCGTCGAATCTCACTGCCAGCAGGAACCCGAGCTTGGACTATGGTTTCAAAGGAGCTGAAATCGCCATGGCTTCCTACTGC
TCCGAGCTCCAGTATCTCGCGAATCCTGTCACGACACATGTCCAGAGCGCGGAGCAGCACAACCAGGACGTGAACTCGTTGG
GATTGATCTCTTCGAGGAAGACCGCCGAAGCTGTGGATATCCTAAAGTTGATGTCTTCCACTTACTTGGTGGGACTTTGTCAA
GCTGTTGACTTGAGGCACTTGGAAGAGAATCTCAGGAGTGCAGTGAAGAACACTGTGAGCCAAGTGGCTAAGAAGGTTTTAA
CAATGGGGTCGAACGGAGAGCTCCACCCATCGAGGTTCTGCGAGAAAGACTTGCTCCAAGTTGTCGATCACGAATACGTGTT
TGCATATGCTGACGATGCTTGCAGCGCAAGCTACCCACTGATGCAGAAGCTGAGACAGGTTTTGGTGGAGCACGCATTGGCT
AATGGCGAGAGTGAGACGAATGCTAGCACTTCAGTGTTCCAAAAGATTGTAGCTTTCGAGGAAGAGCTGAAAACCCTTCTTC
CTAAAGAAGTGGAGAGTGTTAGGGCAGCTTACGAAAGTGGTAAAGCTCCCATCCCTAACAAGATCTTGGAGTGCAGATCTTA
CCCGTTGTACAAATTCGTGAGGGAGGAGCTCGGAACCGCGTTGCTCACCGGAGAGAAAGTTATGTCGCCTGGGGAGGAGTTC
GACAAAGTGTTCACCGCATTGTGCGAGGGGAAGATCATCGATCCGATGATGGACTGCCTCAACGAATGGAATGGTGCCCCTA
TCCCAATCTGCTAAGTTGGTTCACTTATGGAAAATTTGTTATTCCTCTTTTTCATTAATCCCCTTGTTGAATTTGTTGTTATTGTC
TGTCCCAAATCCAAATTTGTAATGGTGTGTAAGTTTGTGATATAAGGGAAAGAAATCCACTTTGTTCCTTCCACTGCTGTTATA
AATTAA

**genolin_c904 coverage by 60-mers probes**
CCTTTAGCAGATTGGGATAGGGGCACCATTCCATTCATGGTGGCAACTTCCAGGGAACTCCCMTYGGRGTGTCGATGGACAA
TGTCCGGTTGGCGATTGCTTCGATCGGGAAGTTAATGTTCGCTCAGTTCAGCGAGCTAGTCAATGATTTCTACAACAATGGAC
TGCCGTCGAATCTCACTGCCAGCAGGAACCCGAGCTTGGACTATGGTTTCAAAGGAGCTGAAATCGCCATGGCTTCCTACTGC
TCCGAGCTCCAGTATCTCGCGAATCCTGTCACGACACATGTCCAGAGCGCGGAGCAGCACAACCAGGACGTGAACTCGTTGG
GATTGATCTCTTCGAGGAAGACCGCCGAAGCTGTGGATATCCTAAAGTTGATGTCTTCCACTTACTTGGTGGGACTTTGTCAA
GCTGTTGACTTGAGGCACTTGGAAGAGAATCTCAGGAGTGCAGTGAAGAACACTGTGAGCCAAGTGGCTAAGAAGGTTTTAA
CAATGGGGTCGAACGGAGAGCTCCACCCATCGAGGTTCTGCGAGAAAGACTTGCTCCAAGTTGTCGATCACGAATACGTGTT
TGCATATGCTGACGATGCTTGCAGCGCAAGCTACCCACTGATGCAGAAGCTGAGACAGGTTTTGGTGGAGCACGCATTGGCT
AATGGCGAGAGTGAGACGAATGCTAGCACTTCAGTGTTCCAAAAGATTGTAGCTTTCGAGGAAGAGCTGAAAACCCTTCTTC
CTAAAGAAGTGGAGAGTGTTAGGGCAGCTTACGAAAGTGGTAAAGCTCCCATCCCTAACAAGATCTTGGAGTGCAGATCTTA
CCCGTTGTACAAATTCGTGAGGGAGGAGCTCGGAACCGCGTTGCTCACCGGAGAGAAAGTTATGTCGCCTGGGGAGGAGTTC
GACAAAGTGTTCACCGCATTGTGCGAGGGGAAGATCATCGATCCGATGATGGACTGCCTCAACGAATGGAATGGTGCCCCTA
TCCCAATCTGCTAAGTTGGTTCACTTATGGAAAATTTGTTATTCCTCTTTTTCATTAATCCCCTTGTTGAATTTGTTGTTATTGTC
TGTCCCAAATCCAAATTTGTAATGGTGTGTAAGTTTGTGATATAAGGGAAAGAAATCCACTTTGTTCCTTCCACTGCTGTTATA
AATTAA

**genolin_c31544 coverage by 25-mers probes**
AGTTGCAGCCGAAAGAAGGGCTTGCTCTTGTCAATGGTACAGCTGTTGGATCTGGTTTGGCATCCATGGTTCTGTTCGAAGCC
AATGTTCTTTCTGTACTGTCTGAGATTTTATCAGCCATTTTCGCTGAGGTTATGAACGGGAAGCCAGAGTTCACTGACCATTTG
ACTCACAAGTTGAAGCATCAGTCCGGGGCAGTA

**genolin_c31544 coverage by 60-mers probes**
AGTTGCAGCCGAAAGAAGGGCTTGCTCTTGTCAATGGTACAGCTGTTGGATCTGGTTTGGCATCCATGGTTCTGTTCGAAGCC
AATGTTCTTTCTGTACTGTCTGAGATTTTATCAGCCATTTTCGCTGAGGTTATGAACGGGAAGCCAGAGTTCACTGACCATTTG
ACTCACAAGTTGAAGCATCAGTCCGGGGCAGTA

**genolin_c5187 coverage by 25-mers probes**
ATGTCAACTCATTAGGGGACTGATTTCGGTTTCTTAGGTAAGACTGCCGAAGCTGTGGACATATTAAAGCTGATGTCGACAA
CTTACTTGGTCGCTCTATGCCAAGCGGTCGACTTAAGGTATATCGAGGAGAATCTGAAGCAGACAGTTAAGAACACGATCAG
CCAAGTAGCGAAGAAAGTACTCCTAACCAACGGGGAGGTGCTCCACCCATCGAGATTCTGCGAGAAGGAGCTATTGAATGTC

GCGGAGAGGGAGTACTTGTTTGCATATGCTGACGATCCTTGCAGCGCAACCTACCCTCTGATGCAGAAGCTGAGAGGGGTGC
TGGTGGAGCACGCGTTGCTGAACGGTGAGAGCGAGAAGGACTCGAGCACTTCCATCTTCCACAAGATTGCAGCTTTTGAGGC
GGAGCTGAAGGCTATCCTGCCGAAAGAAGTCGAGAATGCGAGGAGCGAGCTAGAGAATGGGAACCCGGCTATTCCGAACAG
GATTAAAGAGTGCAGGTCGTATCCGTTGTACAAGTTTGTGAGGGAGGAAGTTGGTACCGGTTTGCTGACGGGTGAGAAGGTT
AAGTCACCTGGTGAGGAATTTGACAAGGTGTTCACTGCTATGTGTGAAGGGAAGCTGATTGATCCTTTGTTGGAGTGCCTCAA
AGAATGGAATGGTGCTCCTCTTCCTATCTGCTAGAACAAGACTTATAAGCATGCATTGCTCTGATGTAATCTTTTGTATTTTCC
TGCAAACAAACTATTATTATCTGATGTAAAAGTGAAGTGGTTTTTGGTTCTTGTTTCCTTTCTTTACCCATTAATGTGTAAGCT
ATCAATCAGTGTATCTTCTCTAATTTTGATTGGGAATCCTTTTCTGTGTACTGGTTTCAAGACAAACGGATGAAATCTGTATAC
GGAAGGAAATAATTAATTCTCCTTGTTGCTTGAGG

**genolin_c5187 coverage by 60-mers probes**

ATGTCAACTCATTAGGGGACTGATTTCGGTTTTCTTAGGTAAGACTGCCGAAGCTGTGGACATATTAAAGCTGATGTCGACAA
CTTACTTGGTCGCTCTATGCCAAGCGGTCGACTTAAGGTATATCGAGGAGAATCTGAAGCAGACAGTTAAGAACACGATCAG
CCAAGTAGCGAAGAAAGTACTCCTAACCAACGGGGAGGTGCTCCACCCATCGAGATTCTGCGAGAAGGAGCTATTGAATGTC
GCGGAGAGGGAGTACTTGTTTGCATATGCTGACGATCCTTGCAGCGCAACCTACCCTCTGATGCAGAAGCTGAGAGGGGTGC
TGGTGGAGCACGCGTTGCTGAACGGTGAGAGCGAGAAGGACTCGAGCACTTCCATCTTCCACAAGATTGCAGCTTTTGAGGC
GGAGCTGAAGGCTATCCTGCCGAAAGAAGTCGAGAATGCGAGGAGCGAGCTAGAGAATGGGAACCCGGCTATTCCGAACAG
GATTAAAGAGTGCAGGTCGTATCCGTTGTACAAGTTTGTGAGGGAGGAAGTTGGTACCGGTTTGCTGACGGGTGAGAAGGTT
AAGTCACCTGGTGAGGAATTTGACAAGGTGTTCACTGCTATGTGTGAAGGGAAGCTGATTGATCCTTTGTTGGAGTGCCTCAA
AGAATGGAATGGTGCTCCTCTTCCTATCTGCTAGAACAAGACTTATAAGCATGCATTGCTCTGATGTAATCTTTTGTATTTTCC
TGCAAACAAACTATTATTATCTGATGTAAAAGTGAAGTGGTTTTTGGTTCTTGTTTCCTTTCTTTACCCATTAATGTGTAAGCT
ATCAATCAGTGTATCTTCTCTAATTTTGATTGGGAATCCTTTTCTGTGTACTGGTTTCAAGACAAACGGATGAAATCTGTATAC
GGAAGGAAATAATTAATTCTCCTTGTTGCTTGAGG
