# Supplementary material for: Intra-platform comparison of 25-mer and 60-mer oligonucleotide Nimblegen DNA microarrays
Source: BMC Res Notes. 2013 Feb 4;6:43. doi: 10.1186/1756-0500-6-43 (PMC3608165; doi:10.1186/1756-0500-6-43)
Supplement: Additional file 5 — Primer design for qRT-PCR. [file 1756-0500-6-43-S5.pdf]

Additional file 5. Primer design for qRT-PCR

Gene ID	Primer position	Primer sequence	Unigene annotation
C24118	forward	TATGCGTTCATTGGGACAGA	Calmodulin TaCaM2-1
C24118	reverse	AGGAACTCCGGGAAATCAAT	
C3323	forward	CAAACCACAAACGCCCTACT	Laccase
C3323	reverse	CGGAGAGAAGGGAGGGATAC	
C21991	forward	TTCCGGTGGATTTCAGTGAT	DNA-directed RNA polymerase II, third largest subunit
C21991	reverse	TTTCGCGATACCTTTCCTTG	
C602	forward	GGCCTCCGACATAATCAAGA	Ribulose bisphosphate carboxylase/oxygenase activase 2
C602	reverse	CGTTGACCATCTGGTTGTTG	
C2533	forward	GGGAGTTGTTGCAGGTGTTT	Cellulose synthase
C2533	reverse	ACGGGTAGAGATGGACGATG	
C822	forward	AGGAAGCTCCTGTTGATTGC	WAX2
C822	reverse	CTCTTGGTGTGATCCACTTCC	
C50701	forward	AGGCTTGCTGTCGTCAGTTG	Similar to nucleolar complex 2
C50701	reverse	GACTGAAGATTCTTCTTTGCGAAC	
C57711	forward	GGGGTGGAGCAAAGAACAG	lcl|genolin_c57711 168 nt
C57711	reverse	AAGCAGCCATTGCTCTTTCA	
C29324	forward	CAATCGACGAGCAGTTCTTG	Cupin
C29324	reverse	CCTCTCCTGTTGGCTGAATC	
